# Molecular Analysis of Glutamate Decarboxylases in *Enterococcus avium*

**DOI:** 10.3389/fmicb.2021.691968

**Published:** 2021-09-10

**Authors:** Xinyi Gu, Jiancun Zhao, Rongling Zhang, Ruohan Yu, Tingting Guo, Jian Kong

**Affiliations:** ^1^State Key Laboratory of Microbial Technology, Shandong University, Qingdao, China; ^2^College of Food Science and Engineering, Shandong Agricultural University, Tai’an, China

**Keywords:** gamma-aminobutyric acid (GABA), GAD system, insertion-inactivation, *Enterococcus avium*, acid tolerance, glutamate decarboxylase

## Abstract

*Enterococcus avium* (*E. avium*) is a common bacterium inhabiting the intestines of humans and other animals. Most strains of this species can produce gamma-aminobutyric acid (GABA) *via* the glutamate decarboxylase (GAD) system, but the presence and genetic organization of their GAD systems are poorly characterized. In this study, our bioinformatics analyses showed that the GAD system in *E. avium* strains was generally encoded by three *gadB* genes (*gadB1*, *gadB2*, and *gadB3*), together with an antiporter gene (*gadC*) and regulator gene (*gadR*), and these genes are organized in a cluster. This finding contrasts with that for other lactic acid bacteria. *E. avium* SDMCC050406, a GABA producer isolated from human feces, was employed to investigate the contribution of the three *gadB* genes to GABA biosynthesis. The results showed that the relative expression level of *gadB3* was higher than those of *gadB1* and *gadB2* in the exponential growth and stationary phases, and this was accompanied by the synchronous transcription of *gadC*. After heterologous expression of the three *gadB* genes in *Escherichia coli* BL21 (DE3), the *K*_m_ value of the purified GAD3 was 4.26 ± 0.48 mM, a value lower than those of the purified GAD1 and GAD2. Moreover, *gadB3* gene inactivation caused decreased GABA production, accompanied by a reduction in resistance to acid stress. These results indicated that *gadB3* plays a crucial role in GABA biosynthesis and this property endowed the strain with acid tolerance. Our findings provided insights into how *E. avium* strains survive the acidic environments of fermented foods and throughout transit through the stomach and gut while maintaining cell viability.

## Introduction

Gamma-aminobutyric acid (GABA), a non-protein amino acid, has several important physiological effects in human including anti-anxiety effects, anti-hypertension effects, anti-inflammatory effects and growth-promoting effects (Li and Cao, 2010; Dhakal et al., 2012; Shin et al., 2014; Bajic et al., 2020; Yilmaz and Gokmen, 2020). Glutamate decarboxylase (GAD) is a key enzyme in GABA synthesis and is widely distributed among animals, plants, and microorganisms (Li et al., 2010). Lactic acid bacteria (LAB) are important GABA producers and have been isolated from fermented foods enriched with GABA (Shin et al., 2014), and some species are part of the normal intestinal microbiome (Walter et al., 2000; Hill and Artis, 2010). In this organ, they can convert dietary glutamate to GABA, thereby providing health benefits to the host (Wu and Shah, 2017). Several *Enterococcus avium* strains have recently been isolated from various fermented foods, particularly East Asian fermented foods, and these strains display a high conversion rate from monosodium glutamate (MSG) to GABA, suggesting that they have the potential to be the starter organisms for GABA-rich functional food production (Tamura et al., 2010; Yang et al., 2016; Lee et al., 2017; Jo et al., 2019). Although a rare pathogen, *E. avium* is often present as part of the normal microbiota in the gastrointestinal tract of individuals, including infants (Birri et al., 2010; Yang et al., 2016). However, to date, the molecular organization of the GAD system in *E. avium* remains unclear, and its functional analysis is lacking.

To cope with acid stress, LAB and other bacterial species employ a variety of acid resistance mechanisms, including the acid tolerance response (ATR) and acid resistance (AR) systems (De Biase and Pennacchietti, 2012; Feehily et al., 2013). The ATR system requires pretreatment of log- or stationary-phase bacteria to mildly acidic pH before acid challenge at pH > 3.0 (Corcoran et al., 2008; De Biase and Pennacchietti, 2012; Scala et al., 2019). The AR system mainly participates in extreme acid stress (pH < 2.5), and its effectiveness relies on GAD, arginine deiminase, urease system, and other amino acid decarboxylases (Cotter et al., 2001; Feehily et al., 2013). The mild acidic environment positively induces the transcription of the GAD system (Cotter et al., 2001; De Biase and Pennacchietti, 2012; Lyu et al., 2018). The proton-consuming decarboxylation of glutamate to GABA, which is then exported out of the cell, increases bacterial tolerance to acid stress while maintaining cell viability in acidic environments (Small and Waterman, 1998; Shin et al., 2014; Krumbeck et al., 2016; Yunes et al., 2016; Lyu et al., 2018; Bajic et al., 2019; Gong et al., 2019). Therefore, the GAD system plays an important role in resistance to acid stress in many types of bacteria (De Biase et al., 1999; Cotter et al., 2005; De Biase and Pennacchietti, 2012; Feehily et al., 2014; Gong et al., 2020).

**FIGURE 1:**
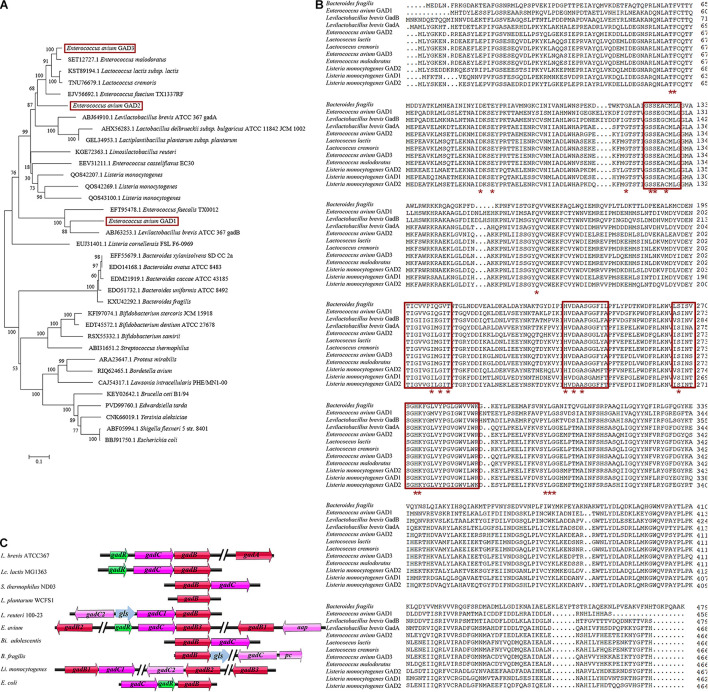
Genetic organization of the glutamate decarboxylase (GAD) system in *E. avium*. **(A)** Phylogenetic tree (maximum-likelihood method) based on the amino acid sequences of GADs from *E. avium* and other GABA-producing species. The GenBank accession numbers of GADs from each strain were indicated. **(B)** Amino acid sequence alignment of three GADs from *E. avium* and other GABA-producing species. The PLP-binding domain was indicated by the red box. The active site residues were marked by the red asterisk. **(C)** Representations of the GAD system in *E. avium* and other GABA-producing species. The *gls* gene encoded glutaminase; the *pc* gene encoded potassium channel; and the *aap* gene encoded amino acid permease, Na/Pi cotransporter, or extra glutamate/GABA antiporter.

In LAB, the GAD system usually comprises the GAD-encoding *gadB* gene, the glutamate/GABA antiporter GadC-encoding *gadC* gene, and the GadR transcriptional regulator-encoding *gadR* gene, all of which are located in the *gad* operon of bacterial genomes, including *Lactococcus lactis* (*Lc. lactis*) (Lyu et al., 2018; Cui et al., 2020; Yogeswara et al., 2020). However, the genetic organization of the GAD system shows species and strain specificity in LAB and other bacterial species (Figure 1A; Gong et al., 2019). Notably, *Levilactobacillus brevis* (*L. brevis*) contains two distinct GAD-encoding genes (*gadA* and *gadB*) and an intact *gad* operon (*gadRBC*) (Wu and Shah, 2017; Wu et al., 2017). The *gadR* gene is missing in the *gad* operon in *Streptococcus thermophilus* (*S. thermophilus*) and *Bifidobacterium adolescentis* (*Bi. adolescentis*) (Yunes et al., 2016; Wu and Shah, 2017). The GAD system of *Lactiplantibacillus plantarum* (*L. plantarum*) consists of only one *gadB* gene and no *gadC* gene (Cui et al., 2020). Interestingly, the glutaminase *gls* gene from *Lactobacillus reuteri* 100-23 is located between two *gadC* genes and participates in the GAD system (Li et al., 2020). The *gadC* gene is next to the potassium channel-encoding *pc* gene and located further downstream of the cluster *gadB*/*gls* in *Bacteroides fragilis* (*B. fragilis*) (Otaru et al., 2021). Unlike the above species, *Listeria monocytogenes* (*L. monocytogenes*) even possesses three completely different *gadB* genes along with two *gadC* genes (Feehily et al., 2014). Similarly, previous research has shown that *E. avium* 352 also possesses three *gadB* genes; however, their contribution to the GAD system is limited (Cui et al., 2020).

In this study, we investigated *E. avium* SDMCC050406, a strain isolated from human feces. This GABA-producing bacterium releases a high level of GABA in growth medium supplemented with MSG, and it carries three *gadB* genes in its genome. We adopted this strain as a model to characterize the contribution of its three *gadB* genes to GABA biosynthesis. We employed bioinformatic and reverse transcription PCR (RT-PCR) analyses and inactivated the *gadB* gene to investigate the contribution of the *gadB* genes in GABA biosynthesis in *E. avium*.

## Materials and Methods

### Bacterial Strains and Culture Conditions

The bacterial strains and plasmids used in this study are listed in Table 1. GABA-producing *E. avium* SDMCC050406 was grown statically in de Man, Rogosa, and Sharpe (MRS) (Oxoid, Basingstoke, United Kingdom) medium at 37°C. For cloning and protein expression purposes, *Escherichia coli* DH5α and *E. coli* BL21(DE3) were grown in Luria–Bertani (LB) medium aerobically at 37°C on a rotary shaker at 200 rpm. *Lc. lactis* MG1363 was grown statically in M17 medium (Oxoid, Basingstoke, United Kingdom) supplemented with 0.5% (wt/vol) glucose (GM17) at 30°C. If necessary, antibiotics (Sangon, China) were added at final concentrations of 5 μg/ml of erythromycin for *Lc. lactis* MG1363 and *E. avium* SDMCC050406 and 100 μg/ml ampicillin or 30 μg/ml kanamycin for *E. coli* DH5α and *E. coli* DE3 (DE3), respectively. MRS medium supplemented with 1% (w/v) MSG (GMRS) assessed GABA production by *E. avium* SDMCC050406.

**TABLE 1:** Bacterial strains and plasmids used in this study.

Strain or plasmid	Characteristics	Sources or references
**Strains**		
*E. avium* SDMCC050406	Wild-type strain isolated from human fecal	This study
*E. avium* SDMCC050406Δ*gadB3*	*gadB3* inactive in *E. avium* SDMCC050406	This study
*Lc. lactis* MG1363	Plasmid-free and prophage-cures derivative of *Lc. lactic* NCDO 712	Gasson, 1983
*E. coli* DH5α *E. coli* BL21 (DE3)	Cloning host Expression host	Novagen Novagen
*E. coli* BL21/pET-Duet1-B1	BL21 containing pET-Duet1-B1	This study
*E. coli* BL21/pET-22b-B2	BL21 containing pET-22b-B2	This study
*E. coli* BL21/pET-28a-B3	BL21 containing pET-28a-B3	This study
**Plasmids**		
pG^+^host9	Erm^r^, integration vector, thermosensitive replicative plasmid in LAB	Biswas et al., 1993
pG^+^host9-Gad	Erm^r^, pG^+^host9 derivative, with the internal fragment of the *gadB3* gene	This study
pET-22b	Amp^r^, expression vector	Novagen
pET-28a	Kan^r^, expression vector	Novagen
pET-Duet1	Amp^r^, expression vector	Novagen
pET-22b-B2	Amp^r^, pET-22b derivative, expression GAD2	This study
pET-28a-B3	Kan^r^, pET-28a derivative, expression GAD3	This study
pET-Duet1-B1	Amp^r^, pET-Duet1 derivative, expression GAD1	This study

### Determination of Gamma-Aminobutyric Acid Content in the Cultures

The concentration of GABA in the cultures was determined by high-performance liquid chromatography (HPLC) with dansyl-chloride (DNS-Cl) (Sangon, China) derivatization method as described (Huang et al., 2006). Briefly, the cell culture supernatants were added at a final concentration of 10% trichloroacetic acid (TCA) to precipitate the protein. The supernatant was diluted with 0.2 M NaHCO_3_ solution and derivatized with 0.4% DNS-Cl–acetone solution at 30°C for 1 h. Then, the mixture was filtered through a 0.2-μm membrane filter (Sangon, China) as samples used for HPLC. The chromatographic separation was performed with a column (Waters Xbridge BEH300 C18 4.6 × 150 mm) and detection performed at 254 nm. A gradient elution protocol with A (methanol)/B (tetrahydrofuran/methanol/50 mM pH 6.2 sodium acetate, 5:75:420, by vol.) as mobile phase was carried out at a flow rate of 0.9 ml/min (0 min 80% B, 6 min 80% B, 20 min 50% B, 20.1 min 0% B, 27 min 0% B, 27.1 min 80% B, 40 min 80% B) at 28°C. GABA concentration was calculated from the integrated peak area comparing with the standard curve constructed by standard GABA (Sigma, United States) solution.

### Total RNA Extraction and Reverse Transcription PCR Assay

The growth of *E. avium* SDMCC050406 in MRS or GMRS broth was monitored by optical density (OD) at 600 nm and pH. Total RNAs from cells collected at the early exponential growth phase (2 h, OD_600_ = 0.25), exponential growth phase (4 h, OD_600_ = 1.00), and stationary phase (8 h, OD_600_ = 1.75) were extracted using an RNA Simple total RNA kit (Tiangen, Beijing, China) according to the protocols of the manufacturer. Subsequently, the extracted RNA was reverse transcribed to cDNA using the PrimeScript RT-PCR kit (TaKaRa, Japan). RT-PCR was carried out with SYBR Premix *Ex Taq* kit (TaKaRa, Japan) in the qTOWER3G system according to the instructions of the manufacturer. The relative expression levels of the five target genes (*gadB1*, *gadB2*, *gadB3*, *gadC*, and *gadR*) were normalized to the constitutive expression of the 16S rRNA housekeeping gene at the same growth phase and were calculated according to the comparative 2^–ΔΔCt^ method with relative expression level of the same gene at the early exponential phase set as 1.0 (Livak and Schmittgen, 2001). The primers used in this study are listed in Table 2. All experiments including culture, RNA extraction, and RT-PCR assays were performed in triplicate independently.

**TABLE 2:** The primers used in this study.

Primer name	Primer sequence (5′–3′)	Restriction sites	Ligated vector
gadB1 F gadB1 R	AACCCTGACACAGCCGAGAC TCGTTTCAAGTGAACCAAGCA		
gadB2 F gadB2 R	CAGTTATCTAGGTGGAGAAATGC GATCTCAAAATGTCGTGAACG		
gadB3 F gadB3 R	TTGGTTCTTCGGAAGCG CCCAGTATAAGTAATTCCCATG		
gadC F gadC R	TTACTGTTGGCTTCGTGAC ATACCGACCACAAATCCTG		
gadR F gadR R	CTGCCGTAGACATTTGGAC TTAAGCGATAAGCGTGGAG		
16S F 16S R	GTCACTGATGGATGGACCCG ATTGCCGAAGATTCCCTACT		
gadB1-Duet1 F gadB1-Duet1 R	CGCGGATCCGATGCATACAGATTATTTAGAACCGCTCGAGTTAGTGGTGGTGGT GGTGGTGAGATGCGTGATGAATCAAATGA	*Bam*H I *Xho* I	pET-Duet1
gadB2-22b F gadB2-22b R	CGCCATATGCTTTATGGAAAGAAAGAT CCGCTCGAGATGGGTAAATCCATAATTT	*Nde* I *Xho* I	pET-22b
gadB3-28a F gadB3-28a R	CATGCCATGGGCATGTTATATGGAAAAGAA GGCCTCGAGTTAGTGGTGGTGGTGGTGGTGATGAGTAAATCCATATGTT	*Nco* I *Xho* I	pET-28a
GadIna F3 GadIna R4	CGGAATTCTTGGCTAAATACAGTGC CCCAAGCTTAATATCCTCGCACAACA	*Eco*R I *Hin*d III	pG^+^host9
GadEN F1	GGCGGACTTAGGCAGTGAGA		
pG^+^host-F	ACTATAGGGCGAATTGGGT		

### Heterologous Expression and Purification of Three Glutamate Decarboxylases

The *gadB1*, *gadB2*, and *gadB3* genes were cloned by PCR amplification using the corresponding primer pairs (gadB1-Duet1 F and gadB1-Duet1 R for *gadB1*; gadB2-22b F and gadB2-22b R for *gadB2*; gadB3-28a F and gadB3-28a R for *gadB3*). PCR products were digested with the corresponding restrictive enzymes and ligated with the vector pET-Duet1, pET-22b, and pET-28a, generating the recombined plasmid pET-Duet1-B1, pET-22b-B2, and pET-28a-B3, respectively. After transformed into *E. coli* BL21 (DE3), the generated three recombinants were overnight grown at 37°C in LB broth containing 100 μg/ml ampicillin or 30 μg/ml kanamycin. Subsequently, 2 ml overnight cultures were diluted into 100 ml fresh LB broth with the corresponding antibiotics and regrown to an OD_600_ of 0.6, and isopropyl-β-D-1-thiogalactopyranoside (IPTG) was added to the medium at a final content of 0.1 mM for induction for 12 h at 16°C, respectively. Cells were harvested and washed in phosphate-buffered saline (PBS, 137 mM NaCl, 2.7 mM KCl, 10 mM Na_2_HPO_4_, 2 mM KH_2_PO_4_, pH 7.4). Afterward, cells were resuspended in 8 ml binding buffer (20 mM sodium phosphate, 20 mM imidazole, 500 mM NaCl, pH 7.4), disrupted by ultrasonication, and centrifugated to remove the cell debris. The overexpressed GAD proteins were purified from supernatants by Ni-NTA affinity chromatograph. The columns were washed with washing buffer (20 mM sodium phosphate, 40 mM imidazole, 500 mM NaCl, pH 7.4) and the His-tagged proteins were eluted with elution buffer (20 mM sodium phosphate, 500 mM imidazole, 500 mM NaCl, pH 7.4). After that, the proteins were dialyzed with 20 mM sodium phosphate buffer (pH 7.4). The concentration and purity levels of proteins were determined by the NanoDrop 2000/2000c UV–Vis (Thermo Fisher Scientific, United States) at 280 nm with bovine serum albumin as a standard (Supplementary Figure 1). The purified proteins were boiled at 75°C for 5 min as soon as the samples were diluted in the 5 × SDS-loading buffer [250 mM Tris–HCl (pH 6.8), 10% sodium dodecyl sulfate (SDS), 0.5% bromophenol blue, 50% glycerin, and 5% 2-hydroxy-1-ethanethiol], and then they were analyzed by SDS-polyacrylamide gel electrophoresis (SDS-PAGE) using 12% (w/v) acrylamide gel.

### Enzymatic Activity Assay

The GAD activity was determined as described previously (Chang et al., 2017). Briefly, the enzyme reaction was carried out with 450 μl of buffer A [20 mM sodium citrate buffer (pH 4.6), 100 mM MSG, 0.1 mM pyridoxal-5′-phosphate monohydrate (PLP)] and 50 μl of 1 mg/mL purified GAD proteins. After incubation at 45°C for 30 min, the reaction was stopped by adding 500 μl of 0.2 M borate saline buffer (pH 9.0) to ensure that that production of GABA was linear and the consumption of MSG was less than 5%. The GABA content was quantified by HPLC. All the enzymatic reactions were carried out in triplicate. One unit of GAD activity was defined as the GABA amount produced by 1 mg/ml enzyme per min under optimal conditions. For the optimal temperature, the purified GADs were incubated with buffer A for 30 min at various temperatures ranging from 30 to 80°C (pH 4.5). In the same way, the optimal pH was determined with 20 mM sodium citrate buffer at pH 3.0 to 7.0. The kinetic parameters were determined with MSG (1–60 mM) as the substrate under the optimal conditions, respectively. The initial velocity of each MSG concentration was determined by measuring GABA production in the first 10 min of reaction. The kinetic constants were estimated by non-linear regression (enzyme kinetics, Michaelis–Menten) using GraphPad Prism 8.2.1. All experiments were performed in triplicate.

### Inactivation of *gadB3* Gene in *Enterococcus avium* SDMCC050406

The *gadB3* gene was inactivated by the temperature-sensitive pG^+^host9 plasmid containing erythromycin selection marker into the active site (Biswas et al., 1993; Lu et al., 2015; Wang et al., 2016). To construct the integration vector, the 804-bp DNA fragment of the *gadB3* gene was PCR amplified from the genomic DNA of *E. avium* SDMCC050406 with the primer pair GadIna F3 and GadIna R4, subsequently inserted into the vector pG^+^host9. The resulting recombined plasmid pG^+^host9-Gad was introduced into *Lc. lactic* MG1363 by electroporation, and the transformants were selected on the plates containing erythromycin after incubation at 30°C for 24 h. Positive clones were selected, and the recombinant plasmid pG^+^host9-Gad was isolated and transformed into *E. avium* SDMCC050406 again by electroporation. The transformants of *E. avium* SDMCC050406 were grown with erythromycin to an OD_600_ of 0.5, and the temperature of growth shifted from 30 to 37°C (non-permissive temperature for plasmid replication) to continue the incubation for 1 h. A serially diluted solution of the transformant cultures was plated onto MRS plates with erythromycin at 37°C for 24 h. The *gadB3* gene was inactivated by homologous crossing, and the mutant *E. avium* SDMCC050406Δ*gadB3* was further verified by PCR amplification with specialized primers GadEN F1 and pG^+^host-F.

### Cell Survival Under Acidic Conditions

Acid tolerance assay was followed as described with modification (Seo et al., 2015; Gong et al., 2019). Briefly, *E. avium* SDMCC050406 and *E. avium* SDMCC050406Δ*gadB3* were grown in GMRS broth for 12 h when the cells were in the stationary phase and began to synthesize GABA. The cells were collected by centrifugation and washed twice with 50 mM potassium phosphate buffer (pH 7.0), followed by suspension in 50 mM potassium phosphate buffer (pH 7.0) to an OD_600_ of 1.0. The cells were harvested by centrifugation again and resuspended in MRS broth (pH 4.0, 3.5, and 3.0) with or without 10 mM MSG. Incubation samples were collected at 2-h intervals over 12 h and serially diluted 10-fold on the MRS agar plates to monitor bacterial survival. All experiments were performed in triplicate.

### Statistical Analysis

All experiments were performed in triplicate. Statistical analysis was performed using unpaired two-tailed Student’s *t*-tests. *p*-values of <0.05 were considered statistically significant.

## Results

### Organization of the GAD System in *Enterococcus avium*

Eighteen whole genomic sequences from *E. avium* strains (as of May 10, 2020) were retrieved from the GenBank database. From them, 53 GAD-encoding amino acid sequences were downloaded from the sequenced genomes of *E. avium* genomes. Sequence alignments suggested that the GADs fell into three independent GAD groups. GAD1 (458 amino acids; aa), GAD2 (466 aa), and GAD3 (466 aa) are encoded by *gadB1*, *gadB2*, and *gadB3* genes, respectively, (data not shown). The maximum-likelihood phylogenetic tree based on the amino acid sequences of the GADs from *E. avium* and other species shows that a distinct relationship exists among the three GADs from *E. avium* (Figure 1A). The multiple sequence alignment for these GADs also showed that GAD1, GAD2, and GAD3 differ (Figure 1B). The PLP-consensus motif was located by aligning the GAD amino acid sequences and from data available at the National Center for Biotechnology Information (NCBI) [CDD Conserved Protein Domain Family: DOPA_deC_like^1^; CDD Conserved Protein Domain Family: Glu-decarb-GAD (see text footnote 1)] and previous reports (De Biase and Pennacchietti, 2012; Yogeswara et al., 2020). The PLP-binding domains and active site residues in the three *E. avium* GADs can be seen to be highly conserved, implying that all of them possess potential decarboxylation activity (Figure 1B).

The genetic organization analysis showed that the *gadB3* gene is located in a three-gene cluster containing the glutamate/GABA antiporter-encoding *gadC* gene and the GadR transcriptional regulator-encoding *gadR* gene. Thus, we surmised that the three genes were probably part of the same operon (Figure 1C). Conversely, *gadB1* and *gadB2* were found to be located in different genomic regions and were not part of an operon. An *app* gene, which encodes an amino acid permease, Na/Pi cotransporter, or extra glutamate/GABA antiporter, was found next to the *gadB1* gene.

### Transcription Levels of the Genes of the Glutamate Decarboxylase System

**FIGURE 2:**
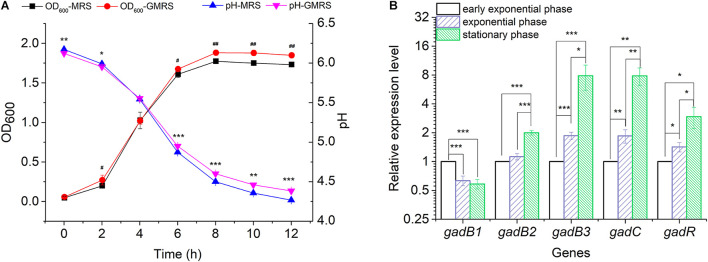
Transcription of the *gadB1*, *gadB2*, *gadB3*, *gadC*, and *gadR*. **(A)** Cell growth curves and pH curves of *E. avium* SDMCC050406 cultivated in MRS or GMRS. Well number represented the statistical significance of OD_600_. Asterisk represented the statistical significance of pH. **(B)** Relative expression levels of *gadB1*, *gadB2*, *gadB3*, *gadC*, and *gadR* in different growth phases. Cells were cultured in GMRS and harvested at early exponential growth phase (2 h), exponential growth phase (4 h), and stationary phase (8 h). The relative expression levels of each gene at early exponential phase were set as 1.0. The relative expression level data were log_2_ transformed. Data were reported as the mean ± SD of the results from three independent experiments. *p-value < 0.05, **p-value < 0.01, ***p-value < 0.001, ^#^*p*-value < 0.05, and ^##^*p*-value < 0.01.

Glutamate Decarboxylase is a key enzyme catalyzing the conversion of glutamate to GABA. To analyze the functional roles played by the three *gadB* genes in GABA biosynthesis, a GABA producer (*E. avium* SDMCC050406) was used to determine the active expression of GABA during the growth of this bacterium in MRS or GMRS broth, respectively. After incubation for 6 h, the bacterial density in GMRS was significantly higher than that in the MRS broth, and this growth was accompanied by an increased pH (Figure 2A). The relative expression levels of *gadB2* and *gadB3* increased along with bacterial growth, achieving ∼2.00- to ∼7.86-fold during the stationary growth phase, respectively, (Figure 2B). With increased *gadB2* and *gadB3* expression levels, the relative transcription levels of *gadC* and *gadR* were also heightened, particularly that of *gadC*, whose improvement was significant. On the other hand, *gadB1* expression decreased with the growth phase, dropping ∼0.58-fold during the stationary growth phase. These results indicated that the three GAD-encoding genes (*gadB1*, *gadB2*, and *gadB3*) were transcribed during the growth phase, and *gadB3* was predominant among them (Figure 2B and Supplementary Figure 2). Furthermore, we confirmed the inducibility of the GAD system by acidity in *E. avium* SDMCC050406 (Supplementary Figure 3). Upon acid treatment, the transcription levels of the three GAD genes increased significantly.

### Biochemical Properties and Kinetic Parameters of the Three Glutamate Decarboxylases

**FIGURE 3:**
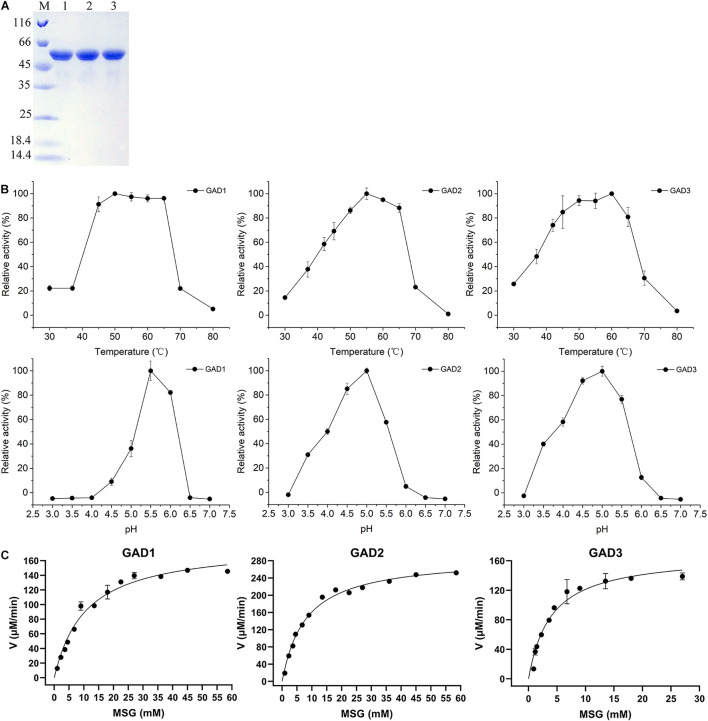
Purification and properties of the recombinant GADs. **(A)** SDS-PAGE of three GADs. Lane M, Pierce^TM^ Unstained Protein MW Marker (Thermo Fisher Scientific, United States), size range shown were 116, 66, 45, 35, 25, 18.4, and 14.4 kDa; lane 1, purified GAD1; lane 2, purified GAD2; lane 3, purified GAD3. **(B)** Effects of temperature and pH value on GAD activities and **(C)** the kinetic parameters of three GADs with MSG as substrate under optimal conditions.

To comparatively analyze the biochemical properties of the three GADs, the *gadB1*, *gadB2*, and *gadB3* genes, whose sizes were 1.377, 1,401, and 1,401 bp, respectively, were cloned from the *E. avium* SDMCC050406 genome. The deduced amino acid sequences were aligned with those from the three aforementioned independent *E. avium* GAD groups. After expression in *E. coli* BL21 (DE3), all three purified GAD proteins were obtained. The UV–visible spectra of the three purified GADs are shown in Supplementary Figure 1. There were only peaks at 280 nm and no obvious PLP peaks at 340 nm. Three GADs were all sized approximately about 55 kDa on SDS-PAGE (Figure 3A), and this agreed with the molecular weights deduced from the amino acid sequences. Interestedly, a minor band lower than 55 kDa by SDS-PAGE was observed in all three GADs but only when samples were boiled at temperatures above 75°C, which is in agreement with the reported anomalous mobilities of proteins on SDS-PAGE (Kurien and Scofield, 2012). Moreover, the biochemical properties of the three GADs differed at various temperatures and pH conditions (Figures 3B,C). The optimal temperature was 50°C for GAD1, 55°C for GAD2, and 60°C for GAD3. The optimal pH was 5.5 for GAD1 and 5.0 for GAD2 and GAD3. Under optimal conditions, *K*_m_ and *V*_max_ were 12.72 ± 1.47 mM and 0.20 ± 0.01 mM/min for GAD1, 8.17 ± 0.99 mM and 0.31 ± 0.01 mM/min for GAD2, and 4.26 ± 0.48 mM and 0.17 ± 0.01 mM/min for GAD3, respectively. Based on these *K*_m_ and *V*_max_ values, the *k*_cat_/*K*_m_ was 28.83 ± 4.87 mM^–1^ s^–1^ for GAD1, 69.56 ± 3.22 mM^–1^ s^–1^ for GAD2, and 73.16 ± 3.75 mM^–1^ s^–1^ for GAD3. Therefore, although GAD3 had the highest MSG preference, its catalytic efficiency was only marginally higher than GAD2 and approximately 2.6 times that of GAD1 (Table 3).

**TABLE 3:** Comparison of properties of three GADs purified from *E. avium* SDMCC050406.

	Predicted molecular weight (kDa)	Optimal temperature (°C)	Optimal pH	*V*_max_ (mM/min)	*K*_m_ (mM)	*k*_*cat*_/*K*_m_ (mM^–1^ s^–1^)
GAD1	55	50	5.5	0.20 ± 0.01	12.72 ± 1.47	28.83 ± 4.87
GAD2	55	55	5.0	0.31 ± 0.01	8.17 ± 0.48	69.56 ± 3.22
GAD3	55	60	5.0	0.17 ± 0.01	4.26 ± 0.48	73.16 ± 3.75

### Inactivation of the *gadB3* Gene and Acid Tolerance Resistance

**FIGURE 4:**
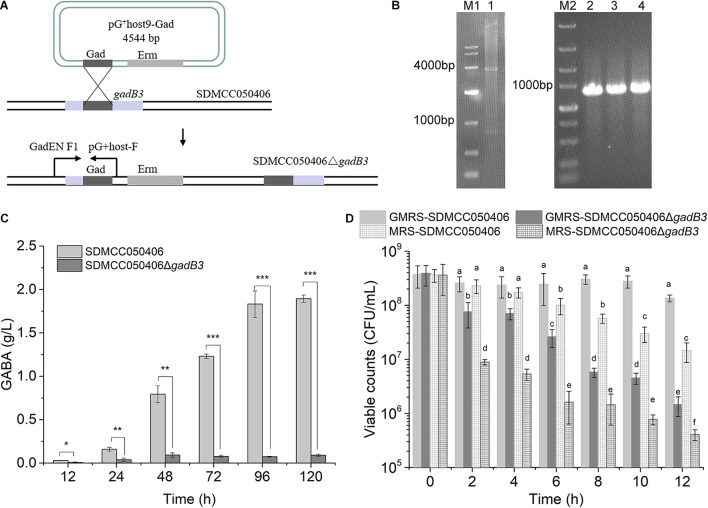
Effect of *gadB3* gene in GABA biosynthesis and acid tolerance. **(A)** Scheme for *gadB3* gene inactivation in *E. avium* SDMCC050406. **(B)** Agarose gel electrophoresis of DNAs, lane M1, DL10,000 DNA Marker (TaKaRa, Japan), size range (10,000, 7,000, 4,000, 2,000, 1,000, 500, and 250 bp); lane 1, the product of pG^+^host9-Gad digested by *EcoR* I and *Hind* III (TaKaRa, Japan); lane M2, DL5,000 DNA Marker (TaKaRa, Japan), size range (5,000, 3,000, 2,000, 1,500, 1,000, 750, 500, 250, and 100 bp); lane 2 to lane 4, PCR products with the primer pair GadEN F1 and pG^+^host-F using the template DNA extracted from three *E. avium* SDMCC050406Δ*gadB3* clones. **(C)** GABA production of *E. avium* SDMCC050406 (black column) and *E. avium* SDMCC050406Δ*gadB3* (gray column) cultured in GMRS. Asterisk represented the statistical significance of GABA. **(D)** Viable cell counts of *E. avium* SDMCC050406 (black line) and *E. avium* SDMCC050406Δ*gadB3* (gray line) under acidic condition of pH 3.5. The cells were harvested at 2-h intervals over 12 h. Error bars represented standard errors from three replicate experiments. The lowercase represented the statistical significance of viable counts. *p-value < 0.05, **p-value < 0.01, and ***p-value < 0.001.

Because *gadB3* displayed the highest relative expression level of the three genes and GAD3 displayed the highest preference for MSG, the *gadB3* gene was inactivated in *E. avium* SDMCC050406 using the temperature-sensitive pG^+^host9 plasmid to investigate the contribution played by the *gadB3* gene in GABA biosynthesis (Figure 4A). The results yielded the mutant *E. avium* SDMCC050406Δ*gadB3* (Figure 4B). To compare the GABA production levels, *E. avium* SDMCC050406 and SDMCC050406Δ*gadB3* were grown in GMRS broth. GABA production in the wild-type SDMCC050406 strain was detected after 12 h of incubation (Figure 4C), the level of which gradually increased along with its growth. When cultured for 120 h, the GABA content in SDMCC050406 reached 1.851 ± 0.205 g/L, whereas only 0.091 ± 0.013 g/L of GABA was detected in SDMCC050406Δ*gadB3* (Figure 4C), indicating that the *gadB3* gene plays a main role in GABA biosynthesis.

Normal GABA production can act to increase bacterial tolerance to acid stress. To further confirm the function of GAD3, as encoded by *gadB3*, the cell survival of *E. avium* SDMCC050406Δ*gadB3* was compared with that of the wild-type SDMCC050406 strain after 12 h of incubation. While the viability of the two strains did not statistically differ at pH 4.0 (data not shown), there was a significant difference at pH 3.5 and 3.0. When subjected to acid stress at pH 3.0, the cell counts for both strains decreased by 4–5 orders of magnitude after 2 h of treatment, which was not dependent on MSG (Supplementary Figure 4). The results of the pH 3.5 test clearly illustrated the role of *gadB3* in acid tolerance (Figure 4D). The viable cell count for the wild-type SDMCC050406 strain was obviously higher than that of the mutant SDMCC050406Δ*gadB3*, both with or without MSG after pH 3.5 treatment, indicating that SDMCC050406Δ*gadB3* cells were more sensitive to acid stress than SDMCC050406 cells (Figure 4D). Therefore, the GAD3 encoded by the *gadB3* gene contributed to bacterial resistance against acidity in *E. avium*.

## Discussion

The GAD system plays important roles in GABA biosynthesis and acid tolerance (De Biase et al., 1999; Cotter et al., 2005; De Biase and Pennacchietti, 2012; Feehily et al., 2014; Gong et al., 2020). Recently, most studies have focused on the distribution and biophysiological function of the GAD system in food-grade lactic acid bacteria (e.g., *L. brevis*, *L. plantarum*, *L. reuteri*, *Lc. lactic*, and *S. thermophilus*), but the GAD system in *Enterococcus* sp., which is highly abundant in the intestinal tract, has rarely been reported. Therefore, this is the first report on the molecular organization of the GAD system and its functional analysis in *E. avium*.

We investigated the gene organization of the GAD system in *E. avium* by bioinformatics analysis (Figure 1C). *E. avium* contains three distinct GAD genes, namely, *gadB1*, *gadB2*, and *gadB3*. These genes clearly differ from those of other GABA-producing species except *L. monocytogenes* (Feehily et al., 2014). Although the organization of the genes of the GAD system in *E. avium* is more similar to that of *L. monocytogenes*, their relationships and amino acid sequences are distinct (Figures 1A,B). The NCBI/Blast database alignments show that GAD1 amino acid sequence shares 83.78% identity with that of *Latilactobacillus curvatus*, GAD2 shares 75.32% identity with that of *Lc. lactis*, and GAD3 shares 90.13% identity with that of *Lc. lactis*. Therefore, the genetic organization of the GAD system in *E. avium* is extremely different from the system of other LAB strains. The distinctiveness of the amino acid sequences of the three GADs implies that their enzymological properties may differ.

The PLP peak at 340 nm (at neutral pH) or 420 nm (at acidic pH) is observed in the spectrum of GADs from *E. coli* and *Brucella microti* (Pennacchietti et al., 2009; Grassini et al., 2015). On the other hand, there was no PLP peak observed in the spectrum of GADs from *E. avium* SDMCC050406. This unexpected and unusual phenomenon may be caused by the presence of the His-tag at the N-terminal end for GAD1 or at the C-terminal end for GAD2 and GAD3 (based on the cloning strategy), which can negatively affect the overall assembly of GAD and its ability to retain PLP (Gut et al., 2006; Grassini et al., 2015). At neutral-alkaline pH, the PLP interacts with a C-terminal His residue and forms the substituted aldamine, which exhibits a characteristic absorption peak at 340 nm (Pennacchietti et al., 2009). Therefore, the presence of the His-tag is likely the cause of the absence of PLP in the three purified GADs. Heterologously expressed GADs from several *Enterococcus* species differ in the conditions required for their optimal activity. Maximal GAD activity was observed at pH 5.0–5.5 and 50–60°C for *E. avium* SDMCC050406 (Figure 3B), pH 5.5 and 45°C for *E. avium* M5, pH 4.8 and 50°C for *Enterococcus faecium* GDMCC60203, and pH 4.6 and 45°C for *Enterococcus raffinosus* TCCC11660 (Chang et al., 2017; Lee et al., 2017; Yang et al., 2020). These pH and temperature values are not the optimal conditions for bacterial growth; therefore, GABA biosynthesis might be affected when these GABA producers are incubated under normal conditions (Wu and Shah, 2017). In addition, although *E. avium* SDMCC050406 GAD3 has a lower *K*_m_ than GAD1 and GAD2 under optimal conditions, *K*_m_ has an intermediate value between that of *E. avium* M5 (3.26 ± 0.21 mM) and *E. raffinosus* TCCC11660 (5.26 μM) (Chang et al., 2017; Lee et al., 2017). These different enzymatic properties might be related to the varied amino acid sequences and conformational structures of these GADs (Wu and Shah, 2017).

In the present study, although *gadB* gene transcription was found to begin during the exponential growth phase (Figure 2B), GABA production was initially detected during the stationary phase (Figure 4C). This indicates that the enzymatic activities of the GADs limit GABA biosynthesis. The optimal growth temperature for *E. avium* SDMCC050406 is 37°C; however, GAD2 and GAD3 exhibit more than 40% enzyme activity at 37°C, whereas GAD1 is less than 20% (Figure 3B). During the stationary growth phase of *E. avium* SDMCC050406, the pH dropped to 4.3 (Figure 2A). GAD2 and GAD3 possess more than 60% enzyme activity at pH 4.0, whereas at this pH, GAD1 enzymic activity abruptly decreased and was almost lost (Figure 3B). GAD3 displays the highest preference for MSG, but its catalytic efficiency is only marginally higher than that of GAD2 and approximately twice that of GAD (Table 3). This suggests that GAD2 and GAD3 are the main enzymatic forms involved in the conversion of glutamate to GABA *in vivo*, and this is particularly true for GAD3.

Due to the gene locus, high transcriptional levels, and optimal enzymatic parameters, GAD3 encoded by *gadB3* was selected to functionally investigate GABA synthesis and acid tolerance in *E. avium* (Figures 1C, 2B, 3B, 4C). The mutant *E. avium* SDMCC050406Δ*gadB3* strain had lower GABA production and viability in acid conditions. Interestingly, a small amount of GABA (0.091 ± 0.013 g/L) was still produced by SDMCC050406Δ*gadB3*, and its slight increase in yield along with its prolonged growth suggests that *gadB1* and *gadB2* genes might functionally substitute for the lack of the *gadB3* gene (Figure 4C). Future studies on knock-out (KO) strains for *gadB1* and *gadB2*, or on the KO strain for *gadB3* complemented with a plasmid expressing *gadB3*, will further confirm the contribution of *gadB3* in GABA production and acid tolerance. In fact, being this the first report on the molecular manipulation of *E. avium*, the production of the KO strains for *gadB1* and *gadB2*, as well as the complementation of the KO strain for *gadB3*, could not be achieved. Nevertheless, our agar gel electrophoresis and sequencing results on *gadB1* and *gadB2* gene PCR products confirmed that *gadB1* and *gadB2* were steadily maintained in the insertion plasmid, thus ruling out the possibility of production of double/triple KO strains.

Although *E. avium* SDMCC050406 produces a low level of GABA (1.851 ± 0.205 g/L, Figure 4C) compared with other the *E. avium* strains isolated from fermented food and plant leaves (Tamura et al., 2010; Lee et al., 2017), as an intestinal isolate, GABA synthesis in this strain could improve bacterial colonization and bacterial survival in the intestinal tract (Figures 4C,D; Small and Waterman, 1998; Shin et al., 2014; Lyu et al., 2018; Gong et al., 2019). The GAD system, which is one of the most efficient bacterial AR mechanisms in withstanding acid stress (Occhialini et al., 2012; Damiano et al., 2015; Wu et al., 2017; Gong et al., 2019), cannot contribute to acid resistance at pH ≤ 3.0 in *E. avium* due to the low catalytic activity of the three GADs in this condition (Figure 3B). Therefore, in the present study, inactivating the *gadB3* gene directly led to a great decrease in GABA production and bacterial survival under acid stress at pH 3.5 (Figures 4C,D). However, the loss of viability from all the strains at pH 3.0 was not dependent on the MSG, further illustrating the weak roles of the GAD system and other anti-acid mechanisms of *E. avium* SDMCC050406 in extremely acidic environments (Supplementary Figure 4). Thus, the GAD system in *E. avium* provides tolerance to acidic environments at pH > 3.0.

In summary, we have detailed the unique distribution of the GAD system genes in *E. avium*, and the *gadB3* gene was experimentally confirmed to be an indispensable factor in GABA biosynthesis. Our findings provide novel insights into the GAD system and GABA biosynthesis in this species.

## Data Availability Statement

The raw data supporting the conclusions of this article will be made available by the authors, without undue reservation.

## Author Contributions

XG and JK contributed conception and design of the study. XG, JZ, RZ, and RY performed the experiments. XG, TG, JZ, and RY performed the statistical analysis. XG, TG, JZ, RZ, and JK wrote and revised the manuscript. All authors read and approved the submitted version.

## Conflict of Interest

The authors declare that the research was conducted in the absence of any commercial or financial relationships that could be construed as a potential conflict of interest.

## Publisher’s Note

All claims expressed in this article are solely those of the authors and do not necessarily represent those of their affiliated organizations, or those of the publisher, the editors and the reviewers. Any product that may be evaluated in this article, or claim that may be made by its manufacturer, is not guaranteed or endorsed by the publisher.

## Footnotes

1
nih.gov

## References

[B1] Bajic S. S. Djokic J. Dinic M. Veljovic K. Golic N. Mihajlovic S. (2019). GABA-producing natural dairy Isolate From artisanal zlatar cheese attenuates gut inflammation and strengthens gut epithelial barrier *in vitro*. *Front. Microbiol.* 10:527. 10.3389/fmicb.2019.00527 30936860 PMC6431637

[B2] Bajic S. S. Dokic J. Dinic M. Tomic S. Popovic N. Brdaric E. (2020). GABA potentiate the immunoregulatory effects of *Lactobacillus brevis* BGZLS10-17 via ATG5-dependent autophagy *in vitro*. *Sci. Rep.* 10:1347. 10.1038/s41598-020-58177-2 31992761 PMC6987229

[B3] Birri D. J. Brede D. A. Forberg T. Holo H. Nes I. F. (2010). Molecular and genetic characterization of a novel bacteriocin locus in *Enterococcus avium* isolates from infants. *Appl. Environ. Microbiol.* 76 483–492. 10.1128/AEM.01597-09 19933345 PMC2805230

[B4] Biswas I. Gruss A. Ehrlich S. D. Maguin E. (1993). High-efficiency gene inactivation and replacement system for gram-positive bacteria. *J. Bacteriol.* 175 3628–3635. 10.1128/jb.175.11.3628-3635.1993 8501066 PMC204764

[B5] Chang C. Zhang J. Ma S. Wang L. Wang D. Zhang J. (2017). Purification and characterization of glutamate decarboxylase from *Enterococcus raffinosus* TCCC11660. *J. Ind. Microbiol. Biotechnol.* 44 817–824. 10.1007/s10295-017-1906-3 28101806

[B6] Corcoran B. M. Stanton C. Fitzgerald G. Ross R. P. (2008). Life under stress: the probiotic stress response and how it may be manipulated. *Curr. Pharm. Des.* 14 1382–1399. 10.2174/138161208784480225 18537661

[B7] Cotter P. D. Gahan C. G. M. Hill C. (2001). A glutamate decarboxylase system protects *Listeria monocytogenes* in gastric fluid. *Mol. Microbiol.* 40 465–475. 10.1046/j.1365-2958.2001.02398.x 11309128

[B8] Cotter P. D. Ryan S. Gahan C. G. Hill C. (2005). Presence of GadD1 glutamate decarboxylase in selected *Listeria monocytogenes* strains is associated with an ability to grow at low pH. *Appl. Environ. Microbiol.* 71 2832–2839. 10.1128/AEM.71.6.2832-2839.2005 15932974 PMC1151821

[B9] Cui Y. Miao K. Niyaphorn S. Qu X. (2020). Production of gamma-aminobutyric acid from lactic acid bacteria: a systematic review. *Int. J. Mol. Sci.* 21:995. 10.3390/ijms21030995 32028587 PMC7037312

[B10] Damiano M. A. Bastianelli D. Al Dahouk S. Kohler S. Cloeckaert A. De Biase D. (2015). Glutamate decarboxylase-dependent acid resistance in *Brucella* spp.: distribution and contribution to fitness under extremely acidic conditions. *Appl. Environ. Microbiol.* 81 578–586. 10.1128/AEM.02928-14 25381237 PMC4277594

[B11] De Biase D. Pennacchietti E. (2012). Glutamate decarboxylase-dependent acid resistance in orally acquired bacteria: function, distribution and biomedical implications of the *gadBC* operon. *Mol. Microbiol.* 86 770–786. 10.1111/mmi.12020 22995042

[B12] De Biase D. Tramonti A. Bossa F. Visca P. (1999). The response to stationary-phase stress conditions in *Escherichia coli*: role and regulation of the glutamic acid decarboxylase system. *Mol. Microbiol.* 32 1198–1211. 10.1046/j.1365-2958.1999.01430.x 10383761

[B13] Dhakal R. Bajpai V. K. Baek K. H. (2012). Production of GABA (γ-aminobutyric acid) by microorganisms: a review. *Braz. J. Microbiol.* 43 1230–1241. 10.1590/S1517-83822012000400001 24031948 PMC3769009

[B14] Feehily C. Finnerty A. Casey P. G. Hill C. Gahan C. G. O’Byrne C. P. (2014). Divergent evolution of the activity and regulation of the glutamate decarboxylase systems in *Listeria monocytogenes* EGD-e and 10403S: roles in virulence and acid tolerance. *PLoS One* 9:e112649. 10.1371/journal.pone.0112649 25386947 PMC4227838

[B15] Feehily C. O’Byrne C. P. Karatzas K. A. (2013). Functional gamma-Aminobutyrate Shunt in *Listeria monocytogenes*: role in acid tolerance and succinate biosynthesis. *Appl. Environ. Microbiol.* 79 74–80. 10.1128/AEM.02184-12 23064337 PMC3536111

[B16] Gasson M. J. (1983). Plasmid complements of *Streptococcus lactis* NCDO 712 and other lactic streptococci after protoplast-induced curing. *J. Bacteriol.* 154 1–9. 10.1128/JB.154.1.1-9.1983 6403500 PMC217423

[B17] Gong L. Ren C. Xu Y. (2019). Deciphering the crucial roles of transcriptional regulator GadR on gamma-aminobutyric acid production and acid resistance in *Lactobacillus brevis*. *Microb. Cell Fact.* 18:108. 10.1186/s12934-019-1157-2 31196094 PMC6567505

[B18] Gong L. Ren C. Xu Y. (2020). GlnR negatively regulates glutamate-dependent acid resistance in *Lactobacillus brevis*. *Appl. Environ. Microbiol.* 86:e02615-19. 10.1128/AEM.02615-19 31953336 PMC7082588

[B19] Grassini G. Pennacchietti E. Cappadocio F. Occhialini A. De Biase D. (2015). Biochemical and spectroscopic properties of *Brucella microti* glutamate decarboxylase, a key component of the glutamate-dependent acid resistance system. *FEBS Open Bio* 5 209–218. 10.1016/j.fob.2015.03.006 25853037 PMC4382515

[B20] Gut H. Pennacchietti E. John R. A. Bossa F. Capitani G. De Biase D. (2006). *Escherichia coli* acid resistance: pH-sensing, activation by chloride and autoinhibition in GadB. *EMBO J.* 25 2643–2651. 10.1038/sj.emboj.7601107 16675957 PMC1478166

[B21] Hill D. A. Artis D. (2010). Intestinal bacteria and the regulation of immune cell homeostasis. *Annu. Rev. Immunol.* 28 623–667. 10.1146/annurev-immunol-030409-101330 20192812 PMC5610356

[B22] Huang J. Mei L. H. Wu H. Lin D. Q. (2006). Biosynthesis of γ-aminobutyric acid (GABA) using immobilized whole cells of *Lactobacillus brevis*. *World J. Microbiol. Biotechnol.* 23 865–871. 10.1007/s11274-006-9311-5

[B23] Jo M. H. Hong S. J. Lee H. N. Ju J. H. Park B. R. Lee J. H. (2019). Gamma-aminobutyric acid production from a Novel *Enterococcus avium* JS-N6B4 strain Isolated from Edible Insects. *J. Microbiol. Biotechnol.* 29 933–943. 10.4014/jmb.1905.05001 31154752

[B24] Krumbeck J. A. Marsteller N. L. Frese S. A. Peterson D. A. Ramer-Tait A. E. Hutkins R. W. (2016). Characterization of the ecological role of genes mediating acid resistance in *Lactobacillus reuteri* during colonization of the gastrointestinal tract. *Environ. Microbiol.* 18 2172–2184. 10.1111/1462-2920.13108 26530032

[B25] Kurien B. T. Scofield R. H. (2012). Common artifacts and mistakes made in electrophoresis. *Methods Mol. Biol.* 869 633–640. 10.1007/978-1-61779-821-4_58 22585529 PMC7295095

[B26] Lee K. W. Shim J. M. Yao Z. Kim J. A. Kim H. J. Kim J. H. (2017). Characterization of a glutamate decarboxylase (GAD) from *Enterococcus avium* M5 Isolated from Jeotgal, a Korean Fermented Seafood. *J. Microbiol. Biotechnol.* 27 1216–1222. 10.4014/jmb.1701.01058 28438014

[B27] Li H. Cao Y. (2010). Lactic acid bacterial cell factories for gamma-aminobutyric acid. *Amino Acids* 39 1107–1116. 10.1007/s00726-010-0582-7 20364279

[B28] Li H. X. Qiu T. Huang G. D. Cao Y. S. (2010). Production of gamma-aminobutyric acid by *Lactobacillus brevis* NCL912 using fed-batch fermentation. *Microb. Cell Fact.* 9:85. 10.1186/1475-2859-9-85 21070676 PMC2996345

[B29] Li Q. Tao Q. Teixeira J. S. Su S.-W. M. Ganzle M. G. (2020). Contribution of glutaminases to glutamine metabolism and acid resistance in *Lactobacillus reuteri* and other vertebrate host adapted *lactobacilli*. *Food Microbiol.* 86:103343. 10.1016/j.fm.2019.103343 31703887

[B30] Livak K. J. Schmittgen T. D. (2001). Analysis of relative gene expression data using real-time quantitative PCR and the 2(-Delta Delta C(T)) Method. *Methods* 25 402–408. 10.1006/meth.2001.1262 11846609

[B31] Lu W. W. Wang Y. Wang T. Kong J. (2015). The global regulator CodY in *Streptococcus thermophilus* controls the metabolic network for escalating growth in the milk environment. *Appl. Environ. Microbiol.* 81 2349–2358. 10.1128/AEM.03361-14 25616791 PMC4357943

[B32] Lyu C. J. Zhao W. R. Peng C. L. Hu S. Fang H. Hua Y. J. (2018). Exploring the contributions of two glutamate decarboxylase isozymes in *Lactobacillus brevis* to acid resistance and γ-aminobutyric acid production. *Microb. Cell Fact.* 17:180. 10.1186/s12934-018-1029-1 30454056 PMC6240960

[B33] Occhialini A. Jiménez de Bagüés M. P. Saadeh B. Bastianelli D. Hanna N. De Biase D. (2012). The glutamic acid decarboxylase system of the new species *Brucella microti* contributes to its acid resistance and to oral infection of mice. *J. Infect. Dis.* 206 1424–1432. 10.1093/infdis/jis522 22930809

[B34] Otaru N. Ye K. Mujezinovic D. Berchtold L. Constancias F. Cornejo F. A. (2021). GABA production by human intestinal *Bacteroides spp.*: prevalence. Regulation, and role in acid stress tolerance. *Front. Microbiol.* 12:656895. 10.3389/fmicb.2021.656895 PMC8082179 33936013

[B35] Pennacchietti E. Lammens T. M. Capitani G. Franssen M. C. John R. A. Bossa F. (2009). Mutation of His465 alters the pH-dependent spectroscopic properties of *Escherichia coli* glutamate decarboxylase and broadens the range of its activity toward more alkaline pH. *J. Biol. Chem.* 284 31587–31596. 10.1074/jbc.M109.049577 19797049 PMC2797229

[B36] Scala G. D. Volonte F. Ricci G. Pedersen M. B. Arioli S. Mora D. (2019). Development of a milk-based medium for the selection of urease-defective mutants of *Streptococcus thermophilus*. *Int. J. Food Microbiol.* 308:108304. 10.1016/j.ijfoodmicro.2019.108304 31425789

[B37] Seo S. W. Kim D. O’Brien E. J. Szubin R. Palsson B. O. (2015). Decoding genome-wide GadEWX-transcriptional regulatory networks reveals multifaceted cellular responses to acid stress in *Escherichia coli*. *Nat. Commun.* 6:7970. 10.1038/ncomms8970 26258987 PMC4918353

[B38] Shin S. M. Kim H. Joo Y. Lee S. J. Lee Y. J. Lee S. J. (2014). Characterization of glutamate decarboxylase from *Lactobacillus plantarum* and its C-terminal function for the pH dependence of activity. *J. Agric. Food Chem.* 62 12186–12193. 10.1021/jf504656h 25415663

[B39] Small P. L. C. Waterman S. R. (1998). Acid stress, anaerobiosis and gadCB lessons from *Lactococcus lactic* to *Escherichia coli*. *Trends Microbiol.* 6 214–216. 10.1016/s0966-842x(98)01285-2 9675796

[B40] Tamura T. Noda M. Ozaki M. Maruyama M. Matoba Y. Kumagai T. (2010). Establishment of an efficient fermentation system of gamma-aminobutyric acid by a lactic acid bacterium, *Enterococcus avium* G-15, Isolated from carrot leaves. *Biol. Pharm. Bull.* 33 1673–1679. 10.1248/bpb.33.1673 20930374

[B41] Walter J. Tannockg W. Tilsala-Timisjarva A. Rodtong S. Loach D. M. Munro K. (2000). Detection and identification of gastrointestinal *Lactobacillus* species by using denaturing gradient gel electrophoresis and species-specific PCR primers. *Appl. Environ. Microbiol.* 66 297–303. 10.1128/aem.66.1.297-303.2000 10618239 PMC91821

[B42] Wang T. Xu Z. S. Lu S. Y. Xin M. Kong J. (2016). Effects of glutathione on acid stress resistance and symbiosis between *Streptococcus thermophilus* and *Lactobacillus delbrueckii* subsp. *bulgaricus*. *Int. Dairy J.* 61 22–28. 10.1016/j.idairyj.2016.03.012

[B43] Wu Q. Shah N. P. (2017). High gamma-aminobutyric acid production from lactic acid bacteria: emphasis on *Lactobacillus brevis* as a functional dairy starter. *Crit. Rev. Food Sci. Nutr.* 57 3661–3672. 10.1080/10408398.2016.1147418 26980301

[B44] Wu Q. Tun H. M. Law Y. S. Khafipour E. Shah N. P. (2017). Common distribution of *gad* operon in *Lactobacillus brevis* and its GadA contributes to efficient GABA synthesis toward cytosolic near-neutral pH. *Front. Microbiol.* 8:206. 10.3389/fmicb.2017.00206 28261168 PMC5306213

[B45] Yang H. Xing R. Hu L. Liu S. Li P. (2016). Accumulation of gamma-aminobutyric acid by *Enterococcus avium* 9184 in scallop solution in a two-stage fermentation strategy. *Microb. Biotechnol.* 9 478–485. 10.1111/1751-7915.12301 26200650 PMC4919989

[B46] Yang S. Y. Liu S. M. Wu Y. Y. Lin Q. Liang G. L. Liu J. F. (2020). Immobilization and enzymatic properties of glutamate decarboxylase from *Enterococcus faecium* by affinity adsorption on regenerated chitin. *Amino Acids* 52 1479–1489. 10.1007/s00726-020-02906-4 33128622

[B47] Yilmaz C. Gokmen V. (2020). Neuroactive compounds in foods: occurrence, mechanism and potential health effects. *Food Res. Int.* 128:108744. 10.1016/j.foodres.2019.108744 31955786

[B48] Yogeswara I. B. A. Maneerat S. Haltrich D. (2020). Glutamate decarboxylase from lactic acid bacteria-A key enzyme in GABA synthesis. *Microorganisms* 8:1923. 10.3390/microorganisms8121923 33287375 PMC7761890

[B49] Yunes R. A. Poluektova E. U. Dyachkova M. S. Klimina K. M. Kovtun A. S. Averina O. V. (2016). GABA production and structure of *gadB*/*gadC* genes in *Lactobacillus* and *Bifidobacterium* strains from human microbiota. *Anaerobe* 42 197–204. 10.1016/j.anaerobe.2016.10.011 27794467

