# Supplementary material for: Molecular Analysis of Glutamate Decarboxylases in Enterococcus avium
Source: Front Microbiol. 2021 Sep 10;12:691968. doi: 10.3389/fmicb.2021.691968 (PMC8461050; doi:10.3389/fmicb.2021.691968)
Supplement: Supplementary file 1 [file Data_Sheet_1.docx]

Supplementary Material

# Supplementary Figures and Tables

## Supplementary Figures


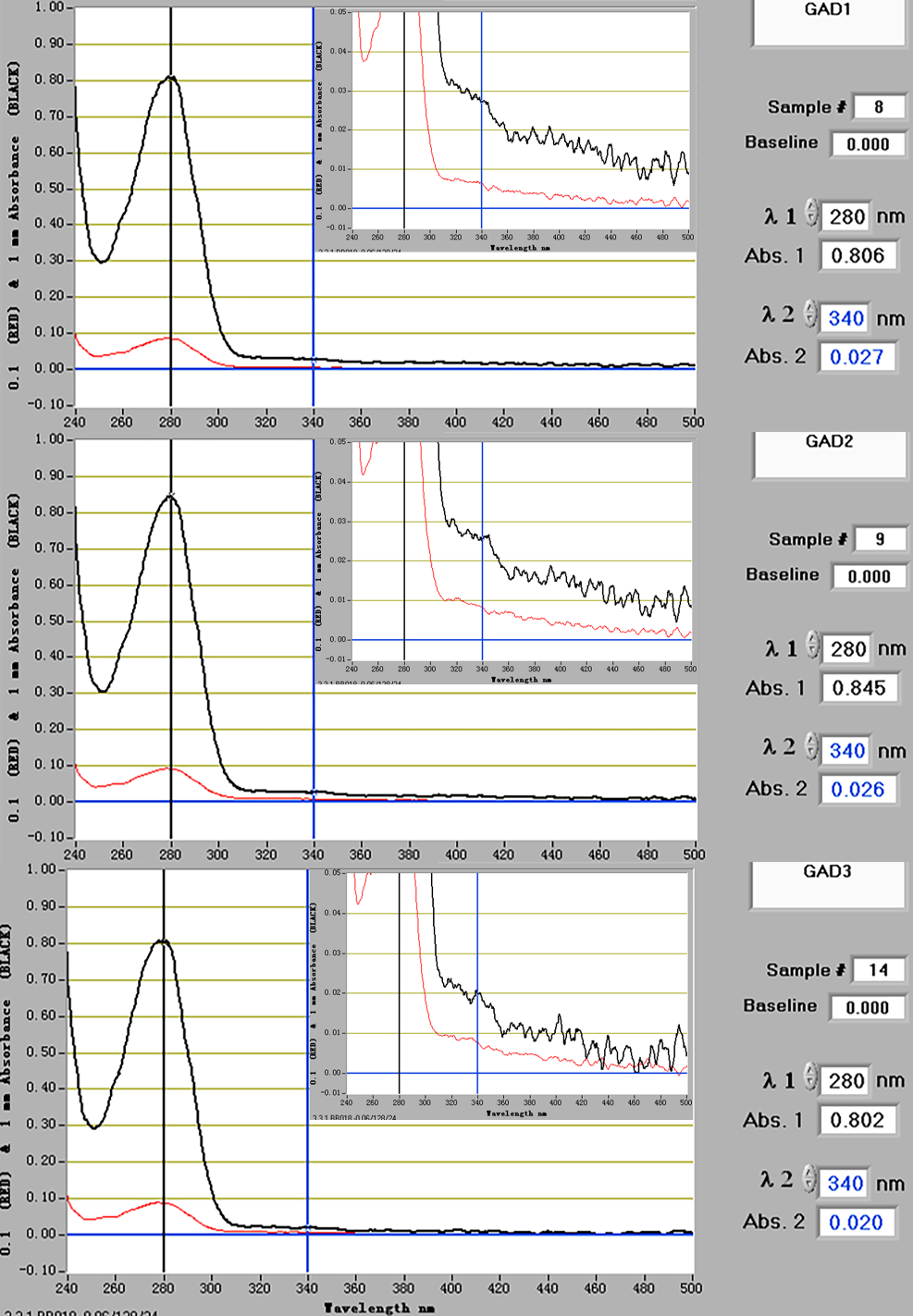


**Supplementary Figure 1. The UV-visible spectra of the three purified GADs.** Three purified GADs in 20 mM sodium phosphate buffer at pH 7.4 were determined by the UV-Vis Spectrophotometer ND-1000 (Nanodrop, USA) at 280 nm and 340 nm with bovine serum albumin as a standard. Inset: zoom of the 0-0.05 absorbance region.


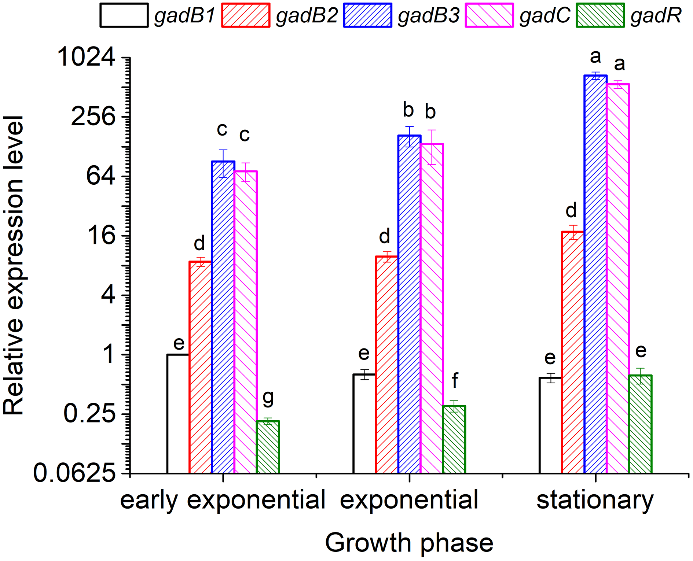


**Supplementary Figure 2. Transcription of the *gadB1*, *gadB2*, *gadB3*, *gadC* and *gadR*.** Relative expression levels of *gadB1*, *gadB2*, *gadB3*, *gadC* and *gadR* at early exponential growth phase (2 h), exponential growth phase (4 h) and stationary phase (8 h). The relative expression level of *gadB1* at early exponential phase was set as 1.0. The relative expression level data were log_2_ transformed. Data were reported as the mean ± SD of the results from three independent experiments. The lowercase represented the statistical significance of the relative expression level.


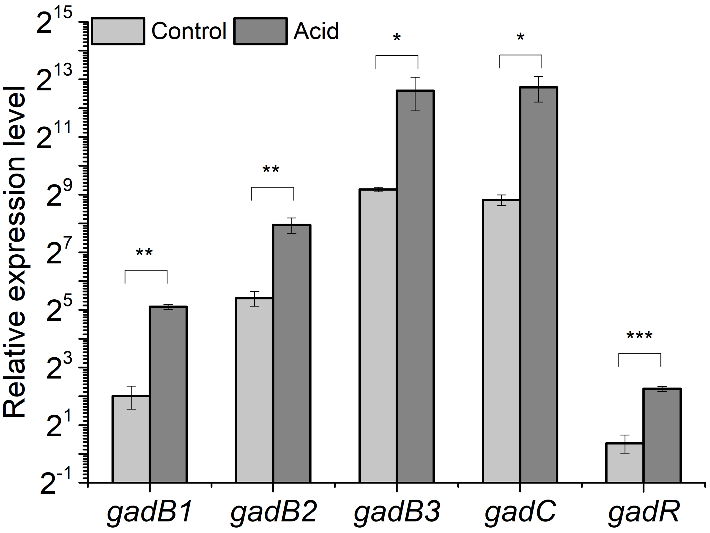


**Supplementary Figure 3. The transcription of the GAD system induced by acidic condition in *E. avium*.** *E. avium* SDMCC050406 was grown in GMRS to an OD_600_ of 0.5 and then the pH was adjusted to 3.5 with 10 M HCl. The control group was not adjusted the pH value. Total RNA was extracted from samples collected after 1 h of incubation. The relative expression level of *gadR* without acid treatment was set as 1.0. The relative expression level of *gadB1*, *gadB2*, *gadB3*, *gadC* and *gadR* with or without acid treatment was shown as the mean ± SD from three independent experiments.


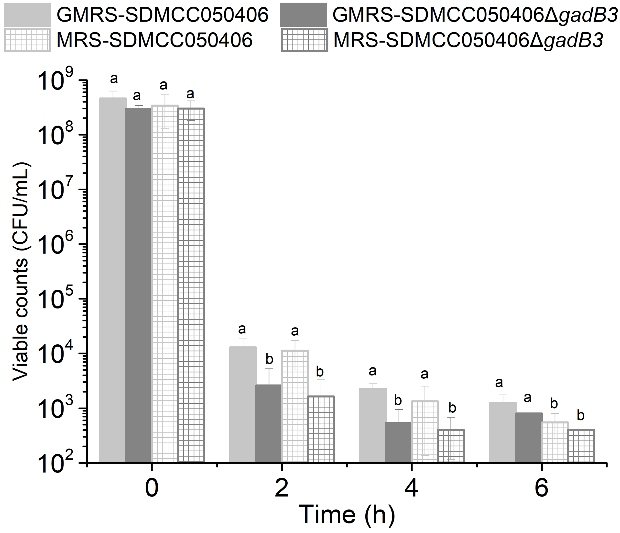


**Supplementary Figure 4. Effect of *gadB3* gene in acid tolerance.** Viable cell counts of *E. avium* SDMCC050406 and SDMCC050406Δ*gadB3* at pH 3.0. The cells were harvested at 2-hours intervals over 6 hours. Error bars represented standard errors from three replicate experiments. The lowercase represented the statistical significance of the viable counts.
